# Calibration of the angle measurement error caused by the industrial reducer performance test instrument torsional deformation

**DOI:** 10.1038/s41598-022-25683-4

**Published:** 2022-12-16

**Authors:** Zhen Yu, Zongrui Hao

**Affiliations:** Institute of Oceanographic Instrumentation, Qilu University of Technology (Shandong Academy of Sciences), 37 Miaoling Road, Qingdao, 266001 China

**Keywords:** Engineering, Mathematics and computing, Nanoscience and technology

## Abstract

The measurement of the stiffness of a precision reducer is essential to estimating the reducer. Since the angular sensor’s measurement results include the angle measurement error caused by the instrument’s torsional deformation, it cannot be used as the actual torsional deformation of the reducer. This paper analyzes the instrument’s torsional deformation characteristics to reduce the angle measurement error. Based on the analysis, a new method of calibrating the angle measurement error based on the improved B-spline curve fitting-gradient descent and particle swarm optimization -radial basis function neural network (IBSCF-GDPSO-RBF) method is proposed. The method can eliminate the angle measurement error caused by the instrument’s torsional deformation. The steps of the IBSCF-GDPSO-RBF method are presented, and the angular measurement error compensation is executed under load conditions. The experiment shows that the instrument deformation caused angle measurement error after compensation is within ± two angular seconds. This paper’s innovation proposes the error calibration method based on the IBSCF-GDPSO-RBF method. It provides a reference for measuring and evaluating the actual torsional rigidity of the Rotary Vector (RV) reducer under any load.

## Introduction

Recently, robot reducers have been widely applied in the automation industry^1^. Significantly, a robot reducer’s characteristics directly affect an industrial robot’s motion accuracy and efficiency^2^. Thus, the robot reducer feature detection significantly benefits the development of the equipment automation sector^3^. Reducer characteristic parameters generally include the starting torque, running torque, and torsional rigidity^4–6^. Multitudinous scholars have extensively studied the reducer’s torsional rigidity and analyzed the reducer’s static characteristics^7–10^. However, these studies are restricted by measurement methods and devices, which cannot promote the improvement of the characteristics of the industrial reducer.

The reducer performance detector is assembled from metal parts rather than an ideal rigid body. In terms of the mechanical structure layout of the whole machine, most detectors adopt a horizontal series structure^11–14^. When the measuring shaft system transmits ample torque, the weak stiffness of a shaft in the instrument shafting will be seriously distorted. Thus, there is a deflection between the accurate torsional deformation of the Rotary Vector (RV) reducer and the angular measurement results. So, it can be seen that the measurement accuracy will be seriously affected by the distortion in the measurement chain in the testing of the reducer’s torsional rigidity. The angular measurement results of the instrument cannot be used as the proper torsional rigidity of the RV reducer^15–17^. A practical method must be adopted to eliminate the effect caused by the torsional deformation of the robot reducer detector^18–20^.

Many experts and scholars have studied this kind of problem. According to the swift effect of large torsional deformation, Wang Zhiqiao et al. theoretically analyzed the deformation angle of a solid circular rod and established the relationship curve between deformation and swift effect^21^. Jia H. K. et al. analyzed the error of the existing torsional deformation measurement methods and gave the calculation formula for angle error^22^. Saygun A. et al. proposed a calculation method for the parts’ torsional stiffness based on finite element analysis^23^. Sigmund O. et al. studied the stress–strain situation of ductile metal materials represented by structural steel after torque, and found that the relationship between stress and strain is linear in a particular range, and the deformation displacement error produced in the process of repeated testing is repetitive^24^. This feature ensures that the angle error caused by metal material deformation is a systematic error, which makes it possible to improve the angle measurement accuracy through a reliable and effective error compensation method. However, all the above research mainly concentrates on the simple deformation of a single part and is not suitable for the complex deformation of the transmission chain in the instrument under high torque.

In order to reduce the angle measurement error, the instrument’s torsional deformation characteristics are analyzed. Based on the features, a new method of calibrating the angle measurement error based on the improved B-spline curve fitting-gradient descent and particle swarm optimization-radial basis function neural network (IBSCF-GDPSO-RBF) method is proposed to eliminate the influence of the instrument torsional deformation. The method is not limited to calibrating the angle measurement error caused by the unavoidable instrument torsional deformation.

The contribution of this paper is that the method calibrates and compensates for the angle measurement error based on the IBSCF-GDPSO-RBF method, which is not limited to measuring the torsional deformation of the RV reducer. The experiment proves that the method can quantitatively detect the proper RV reducer torsional rigidity under any load. It supplies a guideline for measuring and evaluating the proper RV reducer torsional rigidity under any load.

## Analysis of angle measurement error caused by instrument deformation

**Figure 1:**
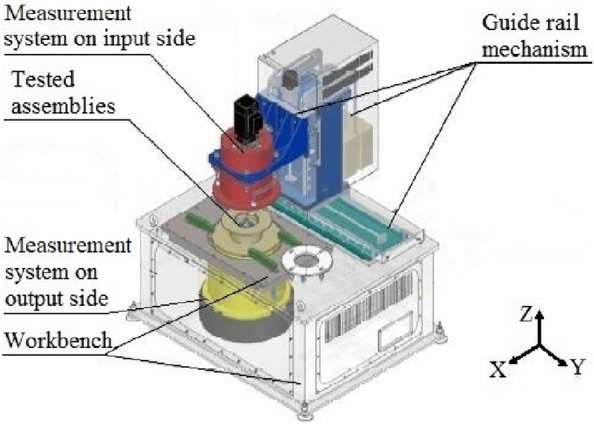
Main structure of the vertical reducer detector.

Relying on the high-precision vertical reducer detector developed by the authors previously^25^, this paper focuses on measuring the torsional rigidity of the RV reducer. The instrument is composed of five subsystems, including a guide rail mechanism, a measurement system on the input side (MSIS), a tested assembly (TA), a measurement system on the output side (MSOS), and a workbench. The overall structure of the instrument is shown in Fig. 1. The external main frame of the instrument adopts a cylindrical structure, and the torque sensor is arranged at the position closest to the input and output end of the tested reducer. This design improves the rigidity of the instrument, simplifies the deformation form of the instrument, shortens the measurement chain, and reduces the number of error sources.

When testing the stiffness of the reducer, the instrument is locked at the MSIS and loaded at the MSOS. The angle measurement system at the MSIS can obtain the torsional stiffness characteristics from its position to the locking device, and the angle measurement system at the MSOS can obtain the torsional stiffness characteristics from its position to the locking device. Then the torsional deformation of the RV reducer is obtained using the measurement results of the angle measurement system at the MSIS minus the measurement results of the angle measurement system at the MSOS, as is shown in formula (1)–(3).

1 $${\theta }_{o}={\theta }_{2}+{\theta }^{{\prime}}+ \theta ,$$

2 $${\theta }_{i}= {\theta }^{{\prime}},$$

3 $${\theta }_{r}={\theta }_{o}-{\theta }_{i},$$

where ${\theta }_{r}$ is the tested torsional deformation of the RV reducer, ${\theta }_{i}$ is the measurement results of the angle measurement system at the MSIS. ${\theta }_{o}$ is the measurement results of the angle measurement system at the MSOS. ${\theta }_{2}$ is the torsional deformation of the shaft between the two angle measurement systems, ${\theta }^{^{\prime}}$ is the torsional deformation of the shaft between the angle measurement system at the MSIS and the locking device, $\theta$ is the actual torsional deformation of the RV reducer.

**Figure 2:**
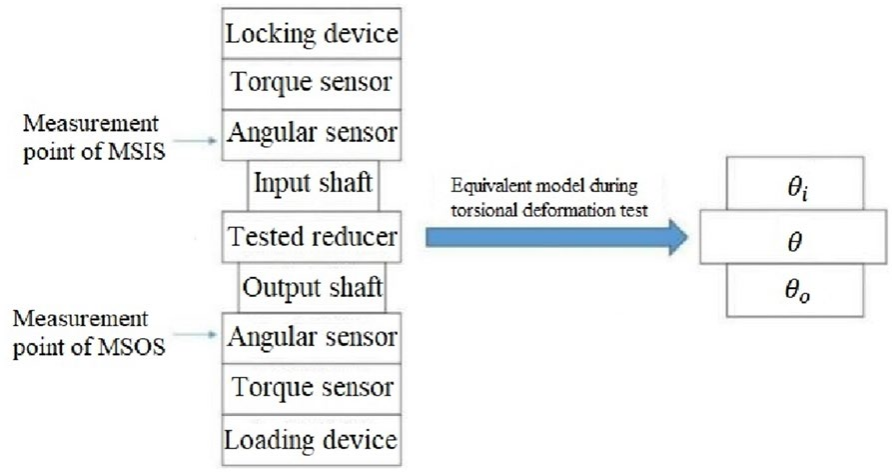
Diagram of the equivalent model during the torsional deformation test of the RV reducer.

It can be seen from the calculation process above that the influence of the unavoidable torsional deformation of the instrument cannot be excluded, as shown in Fig. 2, which means the measurement results include the angle measurement error caused by the torsional deformation of the shaft between the two angle measurement systems.

**Figure 3:**
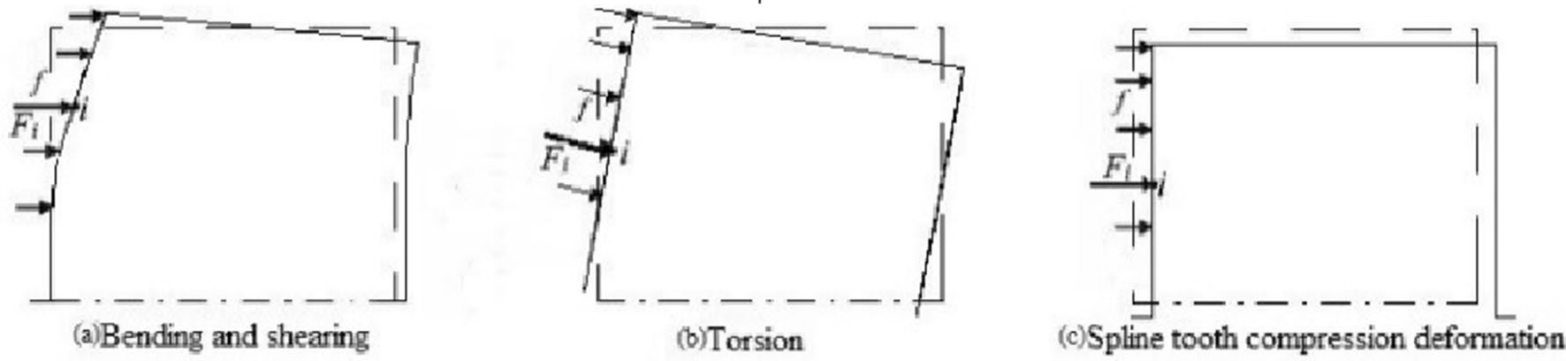
Spline teeth deformation types and characteristics.

**Figure 4:**
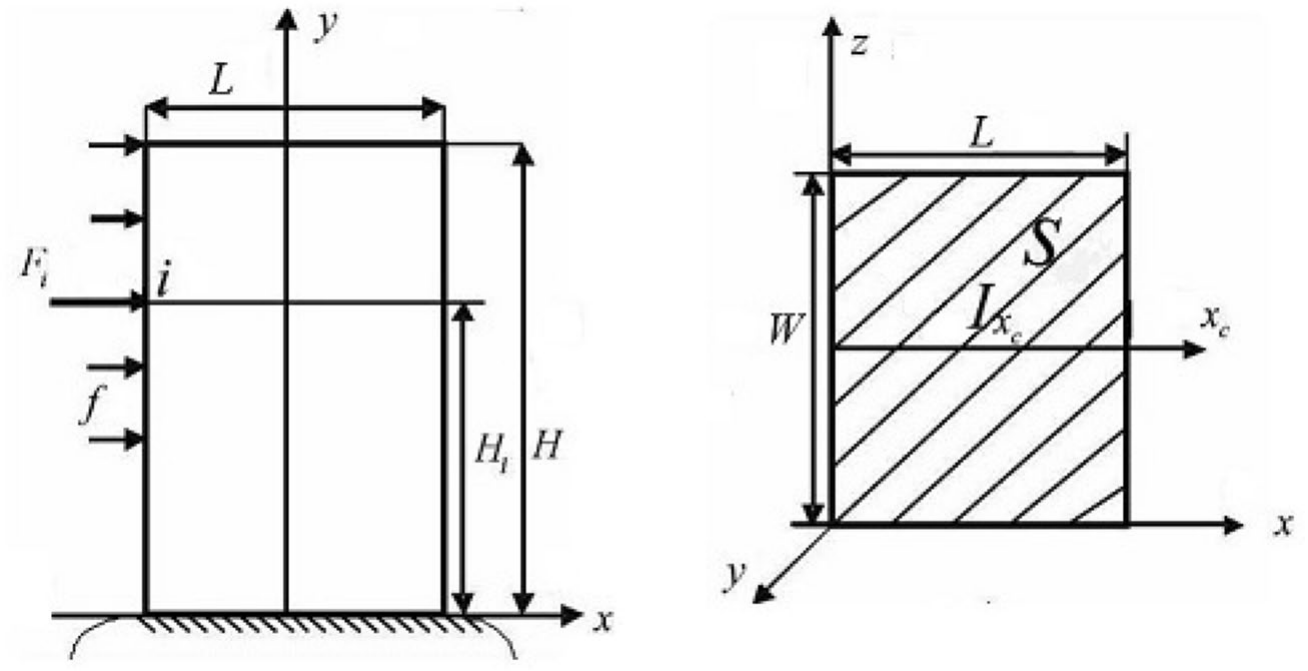
Spline teeth stress analysis diagram.

It can find out from Fig. 2 that the angle measurement error caused by the deformation of the instrument mainly consists of two parts. The first one is the angular measurement error caused by the deformation of the spline coupling in the transmission chain shaft. The second one is the angular measurement error caused by the deformation of the cylindrical platform. All of them are caused by the change in the internal force of the structure. Attention should be paid to the stress–strain characteristics of various shafting parts and the cylindrical bench to ensure that the angle measurement error caused by deformation can be effectively compensated. Considering that the factors leading to the deformation of spline coupling are mainly the combined effect of bending, shearing, torsion, and compression deformation of the spline tooth, the four kinds of deformation are calculated according to the elastic deformation theory of gear teeth^27^. The calculation process adopts the improved integral method. The spline tooth is subdivided into several rectangles by the improved integration method, and the deformation of each rectangle under the action of the uniform force is regarded as the deformation of a cantilever beam under the action of a concentrated force to obtain the deformation components caused by the four cases. The effect of the four types of tooth deformation is shown in Fig. 3, and the force on spline teeth is shown in Fig. 4. The stiffness characteristics of each component in the transmission chain are analyzed in the following section. Furthermore, the angle measurement error model is constructed^26^.

In Fig. 4, f is the force uniformly distributed on the surface of the key tooth, and the equal force of the uniformly distributed force ${F}_{i}$ acts on point $i$, where L is the key width of the rectangular spline tooth, $H$ is the height of the key tooth, and ${H}_{i}$ is the height from the equal force action point $i$ to the root of the key tooth, W is the thickness of the key tooth, S is the cross-sectional area of the key tooth, ${I}_{{x}_{c}}$ is the polar moment of inertia of the cross-section, μ is Poisson’s ratio, and E is elastic modulus.

(1) Angle measurement error caused by shaft deformation.

For the transmission chain shaft described in this paper, the radius is ${r}_{1}$ and the length is ${L}_{1}$. The angular measurement error ${\Delta \theta }_{1}$ caused by cylindrical shaft deformation is^27^:

4 $${\Delta \theta }_{1}=\frac{2T{L}_{1}}{G\pi {r}_{1}^{4}}.$$

In the above formula, $G$ is the material’s shear modulus, and $T$ is the transmitted torque.

(2) Angle measurement error caused by deformation of cylindrical bench.

For the cylindrical bench of the instrument, the primary diameter is ${d}_{1}$ and the minor diameter is ${d}_{2}$. The length of the cylindrical bench, located between the circular grating reading head and the tested reducer, is ${L}_{2}$. The angle measurement error ${\Delta \theta }_{2}$ caused by the deformation of the cylindrical bench is^27^:

5 $${\Delta \theta }_{2}=\frac{32T{L}_{2}}{G\pi \left({d}_{1}^{4}-{d}_{2}^{4}\right)}.$$

In the above formula, $G$ is the material’s shear modulus, and $T$ is the transmitted torque.

(3) Angle measurement error caused by spline tooth bending deformation.

The bending deformation of spline teeth can be equivalent to the deformation of the cantilever beam fixed at one end under external force. The bending moment ${M}_{1}$ of the spline teeth is:

6 $${M}_{1}=\frac{Tcos\alpha \left(H-{H}_{i}\right)}{{r}_{2}}-\frac{Tsin\alpha {W}_{a}}{{r}_{2}}.$$

In the equation above, ${H}_{i}$ is the height from the equal force action point $i$ to the root of the key tooth, ${W}_{a}$ is the half-tooth thickness corresponding to the point of equivalent force, $H$ is the key tooth height, $T$ is the transmitted torque, ${r}_{2}$ is the spline pitch circle radius, $\alpha$ is the angle between the equivalent force and spline radial direction.

The angle measurement error ${\Delta \theta }_{3}$ caused by the bending moment ${M}_{1}$ is:

7 $${\Delta \theta }_{3}=\frac{T[2({cos\alpha )}^{2}{H}^{2}-3sin\alpha cos\alpha {W}_{a}H-3sin\alpha cos\alpha {H}^{2}+6{(sin\alpha )}^{2}{W}_{a}H]}{6{E}_{e}{I}_{{x}_{c}}{r}_{2}},$$

where ${I}_{{x}_{c}}$ is the inertia couple of the intersecting surfaces, ${E}_{e}$ is the equivalent elastic modulus. The formula used to calculate ${E}_{e}$ is as follows:

8 $${E}_{e}=\frac{E}{1-{\mu }^{2}},$$

where $\mu$ is the Poisson’s ratio, and E is the elastic modulus*.* Generally, $E$=210000Mpa, $\mu$= 0.3.

(4) Angle measurement error caused by spline tooth shear deformation.

The shear deformation and bending deformation of spline teeth have a superposition effect. The shear deformation here refers to the displacement change of teeth under the tangential shear force. Angle measurement error ${\Delta \theta }_{4}$ caused by shear deformation from the tooth root to the contact point can be expressed as:

9 $${\Delta \theta }_{4}=\frac{Tcos\alpha }{GS{r}_{2}}.$$

In the equation above, $G$ is the shear modulus, $S$ is the sectional area, and $T$ is the transmission torque. The general formula of $G$ is:

10 $$G=\frac{{E}_{e}}{2\left(1+\mu \right)}.$$

In the equation above, the sectional area $S$ is a rectangle, and its calculation formula is:

11 $$S=2L{W}_{a}.$$

In the equation above, $S$ is the sectional area at the spline contact point, $L$ is the tooth width, and ${W}_{a}$ is the half-tooth thickness at the contact point.

(5) Angle measurement error caused by torsional deformation of spline teeth.

For the angle measurement error caused by the torsional deformation of spline teeth, the most critical consideration is the change of basal body at the tooth’s root. Under the action of bending moment, the calculation formula of spline tooth stiffness is as follows:

12 $${k}_{j}=\frac{2{E}_{e}{S}_{0}{W}_{0}}{5.3}.$$

In the equation above, ${S}_{0}$ is the sectional area at the tooth root, ${W}_{0}$ is the half-tooth thickness at the tooth root.

The angle measurement error ${\Delta \theta }_{5}$ caused by the deformation of the basal body at the tooth root under the effect of the transmitted torque $T$ can be expressed as:

13 $${\Delta \theta }_{5}=\frac{Tcos\alpha \mathrm{H}-{Tsin\alpha W}_{a}}{{k}_{j}{r}_{2}}.$$

(6) Angle measurement error caused by spline tooth compression deformation.

Compression deformation mainly refers to the compression of the basal body at the tooth’s root. The angle measurement error ${\Delta \theta }_{6}$ caused by the combined compression deformation in the radial and tangential directions can be expressed as:

14 $${\Delta \theta }_{6}=\left(\frac{H{(sin\alpha )}^{2}}{{E}_{e}S{r}_{2}}+\frac{H({cos\alpha )}^{2}}{{E}_{e}L{W}_{a}{r}_{2}}-\frac{(H-{W}_{a})({cos\alpha )}^{2}}{{E}_{e}{W}_{a}L{r}_{2}}\right)\sqrt[3]{\frac{16}{9{\pi }^{2}}\frac{T{R}_{2}}{{\left(\frac{1-{v}_{1}^{2}}{\pi {E}_{1}}+\frac{1-{v}_{2}^{2}}{\pi {E}_{2}}\right)}^{2}\left({R}_{1}+{R}_{2}\right)}}.$$

In the equation above, ${\nu }_{1}$ is the Poisson’s ratio of spline sleeve, ${\nu }_{2}$ is the Poisson’s ratio of spline shaft, ${E}_{1}$ is the elastic modulus of the spline sleeve, ${E}_{2}$ is the elastic modulus of the spline shaft, ${R}_{1}$ is the radius of the spline sleeve, ${R}_{2}$ is the radius of the spline shaft, and $T$ is the torque transmitted.

Based on the above six types of angle measurement errors, the total angle measurement error $\Delta \theta$ is:

15 $$\Delta \theta =\sqrt{{{\Delta \theta }_{1}}^{2}+{{\Delta \theta }_{2}}^{2}+{{\Delta \theta }_{3}}^{2}+{{\Delta \theta }_{4}}^{2}+{{\Delta \theta }_{5}}^{2}+{{\Delta \theta }_{6}}^{2}}.$$

It can be figured out that there is a nonlinear relationship between the angle measurement error $\Delta \theta$ and the transmitted torque $T$. Equation (15) does not fully reflect the angle measurement error. It only considers the angle measurement error caused by the deformation of each component in the shaft and does not consider the angle measurement error caused by the combination of spline coupling, compression positioning, and other contact methods used for the shafting connection. The contact stability between components in the shaft under ample torque must be considered in the calibration and compensation of angle measurement error. Therefore, the model formula cannot analyze the angle measurement error caused by the deformation of the shaft. The deformation of the shaft needs to be identified from the angle measurement results, and then the relationship between transmitted torque and angular displacement of the shaft should be further obtained. The following part of this paper focuses on calibrating and compensating for the angle measurement error caused by the deformation of the drive chain shafting in the instrument.

## The calibration method of the angle measurement error caused by instrument deformation

According to the second section analysis, measuring the angle measurement error caused by the shaft’s deformation and obtaining the relationship between the angle measurement error and transmitted torque is necessary. As mentioned above, when the tested reducer is loaded on the instrument, the measurement results of the angular sensor on the instrument will inevitably include the angle measurement error caused by the torsional deformation of the shaft. It is necessary to obtain the comprehensive deformation of the transmission chain at the MSIS and MSOS to improve the measurement accuracy of the stiffness curve of the reducer. The best idea to exclude the influence of the instrument’s deformation is to use an ideal reducer with known stiffness as the standard body. When the reducer with ideal stiffness is located at the measured position, the deformation of the instrument’s components can be obtained using the measurement results of the angle measurement system minus the deformation of the ideal standard reducer. The result is recorded as $\Delta \theta$. It means the angle increment $\Delta \theta$ is caused by the deformation of the instrument’s components. In the test of the deformation of other reducers, it is necessary to eliminate the deformation angular displacement $\Delta \theta$. Then, the characteristics of the tested reducers are analyzed.

**Figure 5:**
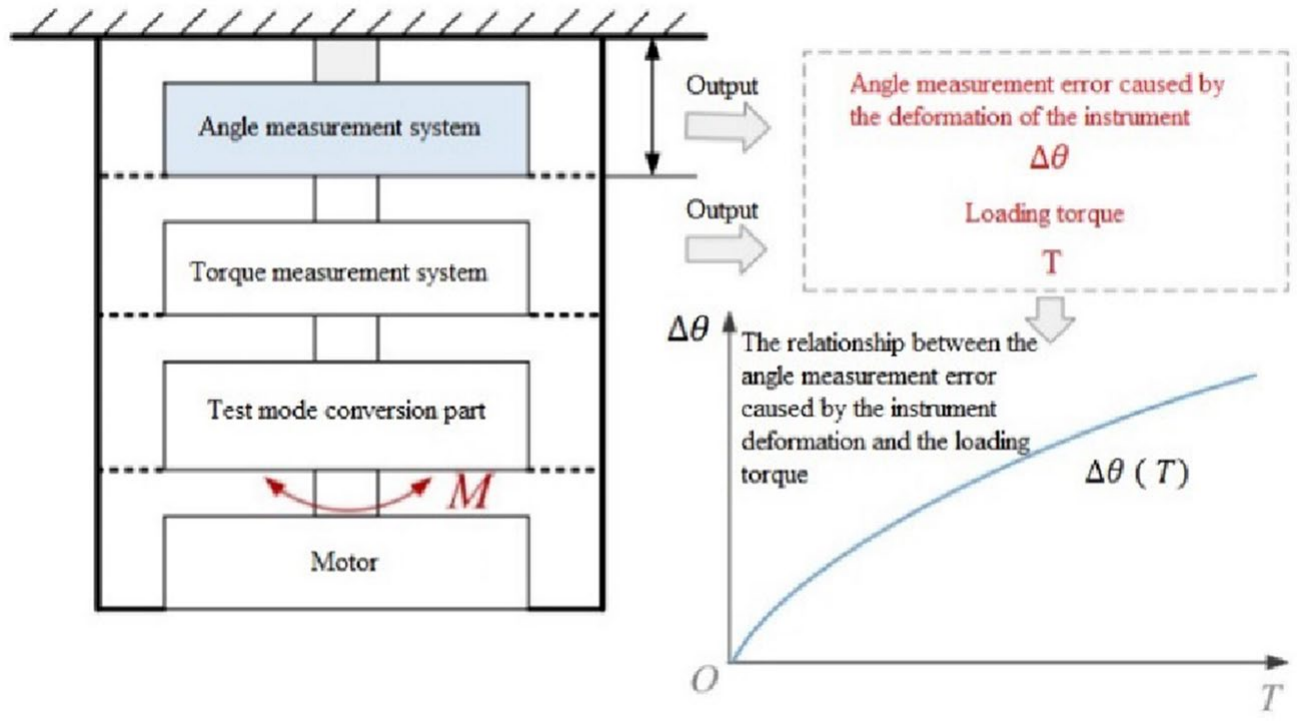
Measurement scheme for the angle measurement error caused by the instrument shaft deformation.

The ideal reducer with known stiffness is difficult to find in practice. The entity with quasi-infinite stiffness can be used to replace the reducer with known ideal stiffness. As shown in Fig. 5, the MSIS (or MSOS) measuring shafts are directly consolidated with the barrel (its stiffness is quasi-infinite). The MSIS (or MSOS) motors apply torque from zero to the full range required for the test. At the same time, the outputs of the angle measuring system and torque measuring system at the MSIS (or MSOS) are recorded, and the “angular displacement-torque” curve is formed. This curve represents the rule of comprehensive deformation of instrument shafts, which can be used to obtain the angle measurement error caused by the deformation of the shafts. This angular displacement in the curve is the angular measurement error value to be compensated during the reducer test. The circular grating system reads the angular displacement, so it just represents the part of the deformation of instrument shafts included in the angle measurement results. The part of the deformation that is not included in the circular grating reading can be ignored because it does not affect the accuracy of the angle measurement of the instrument.

**Figure 6:**
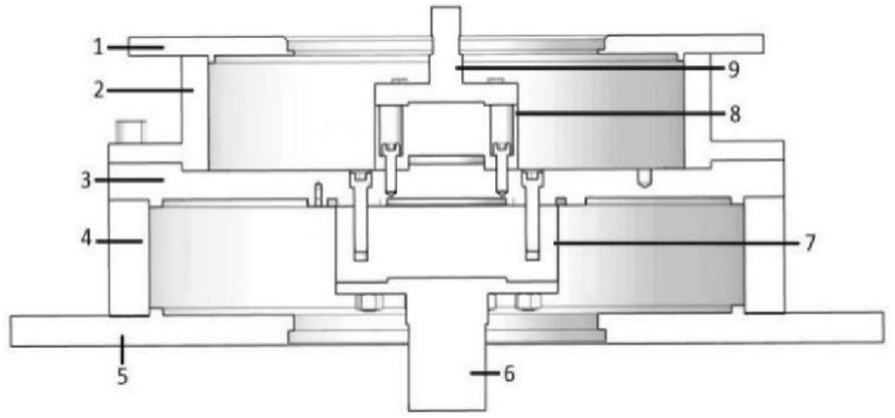
The instrument deformation calibration device. In the figure: 1. Top cover; 2. Input barrel; 3. Intermediate plate; 4. Output barrel; 5. Base; 6. Spline shaft at output side; 7. Spline shaft fixing block at output side; 8. Spline shaft fixing block at input side; 9. Input side spline shaft.

The feature of this idea is that it is not necessary to know the deformation of every component or the contact form between components in the actual measurement process. The angle measurement error caused by the deformation of the shafts can be obtained through the actual measurement. The angular displacement introduced by the measured reducer angular displacement and instrument structure deformation could be identified through this idea. This paper proposes an angle-torque calibration method based on this idea. The instrument deformation calibration device is designed, as shown in Fig. 6. The real-time special compensation for angle measurement error caused by instrument deformation is realized.

The designed instrument deformation calibration device adopts the exact positioning and clamping structure as the TA. Furthermore, it can be installed on the instrument and matched with the MSIS and MSOS mechanical interfaces to calibrate the angle measurement error caused by the deformation of the instrument shafting under specific torque. When the MSIS and MSOS are subject to torque, the readings of the angle measuring system at the MSIS and MSOS reflect the angle measurement error caused by the comprehensive deformation of the respective drive chain shafting and cylindrical workbench at the MSIS and MSOS. Then the angle data under different torques should be recorded, and the angle measurement error list of the measuring system at the MSIS and MSOS should be formed.

**Figure 7:**
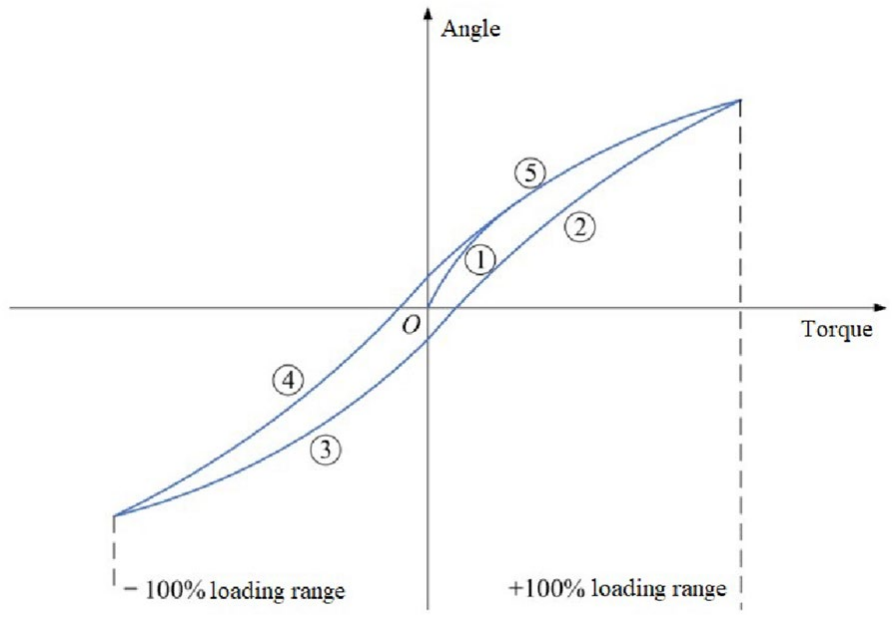
Loading mode of angle error calibration.

**Figure 8:**
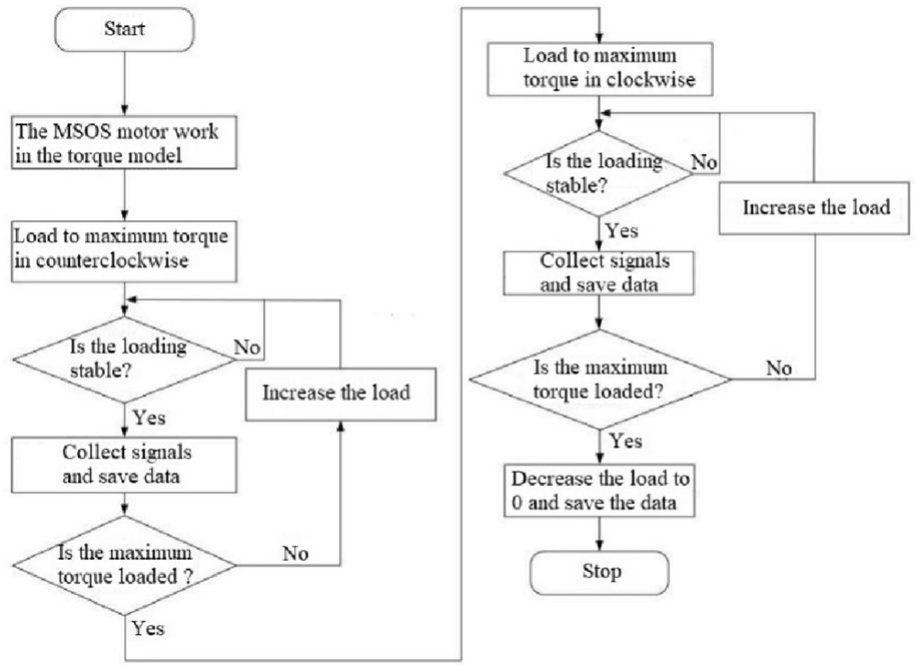
Flow chart of the calibration of angle measurement error.

In the angle measurement error calibration, one-way loading and one-way unloading are adopted to avoid discontinuity and difficulty in fitting. That is, in the order of ④ → ⑤ → ② → ③, as shown in Fig. 7. Therefore, one-way loading and one-way unloading are adopted in the process of angle measurement error calibration, and whether the backlash in the calibration process is lower than the set threshold value is judged. If the backlash is higher than the set threshold value, it is necessary to check whether the fixed state of the shafting is normal. The calibration process of angle measurement error caused by deformation is shown in Fig. 8.

## Compensation method for the angle measurement error caused by instrument deformation

Due to the extensive torque loading range of the instrument and the instability of loading, it is difficult to calibrate the angle measurement error caused by the deformation of the instrument in the whole torque loading range continuously during the calibration process. Because only some torque loading points are calibrated, a specific method must be adopted to realize the real-time compensation of angle measurement error within the entire torque loading range. This paper selects 1000 points evenly distributed in the loading range for angle measurement error calibration. At the same time, the average value filtering method is used to offset the impact of loading stability as much as possible.

In the quasi-static experiment, the angle measurement error caused by the combined deformation of the MSIS and MSOS shafting and the cylindrical worktable is nonlinear with the transmitted torque. Therefore, after obtaining the angle measurement error caused by the deformation of the MSIS and MSOS, the IBSCF-GDPSO-RBF method is used to obtain the angle measurement error-loading torque relationship model. The IBSCF-GDPSO-RBF method combines improved B-spline curve fitting, gradient descent, particle swarm optimization, and radial basis function neural network. The IBSCF-GDPSO-RBF method is used because the improved B-spline curve fitting method can fit any nonlinear function relationship with the smallest residual possible, and the GDPSO-RBF method can approach any function with arbitrary accuracy. The IBSCF-GDPSO-RBF method can be used to make full use of discrete calibration points and realize continuous difference compensation in the measurement range. The method is described in detail below.

### Improved B-spline curve fitting (IBSCF)

Traditional B-spline curve fitting is to fit a curve every four points. The curve does not pass-through type value points but has the advantages of locality, continuity, and convexity. The equation of B-spline curve fitting is:

16 $$r\left(t\right)=\sum_{i=0}^{3}{B}_{i}\left(t\right){P}_{i},$$

where, ${P}_{i}$ is the characteristic polygon vertex, also known as the control vertex. ${B}_{i}\left(t\right)$ is the basis function. The basis function can be expressed as:

17 $${B}_{0}\left(t\right)=\frac{1}{6}\left(-{t}^{3}+3{t}^{2}-3t+1\right),$$

18 $${B}_{1}\left(t\right)=\frac{1}{6}\left(3{t}^{3}-6{t}^{2}+4\right),$$

19 $${B}_{2}\left(t\right)=\frac{1}{6}\left(-3{t}^{3}+3{t}^{2}+3t+1\right),$$

20 $${B}_{4}\left(t\right)=\frac{1}{6}{t}^{3}.$$

According to the characteristics of the B-spline curve fitting endpoint, it is only necessary to make the type value point located at the middle line of the triangle bottom, 1/3 away from the vertex, and the tangent vector of the type value point parallel to the bottom to make the fitted curve pass through the type value point and reduce the error. Therefore, the original type value points can meet the above conditions by adding type value points. Moreover, the starting and end points of the curve are specially treated, which is the basic idea of the IBSCF algorithm.

Assuming that the number of original type value points is n, 3n-3 B-spline curves can be obtained by increasing the number of type value points to 3n, and all original type value points can be passed. The new value point coordinates calculation method is given below, excluding the first and last endpoints.

21 $${A}_{i,0}={P}_{i}+{P}_{i}{A}_{i,0}={P}_{i}-h\left({P}_{i+1}-{P}_{i-1}\right),$$

22 $${A}_{i,0}={P}_{i}+{P}_{i}{A}_{i,1}={P}_{i}+h\left({P}_{i+1}-{P}_{i-1}\right),$$

According to Eqs. (16)–(20), the three continuous fitting curves can be expressed in matrix form as:

23 $${r}_{3i-2}\left(t\right)=\frac{1}{6}\left[\begin{array}{ll}\begin{array}{ll}{t}^{3}& {t}^{2}\end{array}& \begin{array}{ll}t& 1\end{array}\end{array}\right] \left[\begin{array}{*{20}c} { - 1} & 3 & { - 3} & 1 \\ 3 & { - 6} & 3 & 0 \\ { - 3} & 0 & 3 & 0 \\ 1 & 4 & 1 & 0 \\ \end{array}\right] \left[\begin{array}{l}{A}_{i,0}\\ {P}_{i}\\ \begin{array}{l}{A}_{i,1}\\ {A}_{i+1,0}\end{array}\end{array}\right],$$

24 $${r}_{3i-1}\left(t\right)=\frac{1}{6}\left[\begin{array}{ll}\begin{array}{ll}{t}^{3}& {t}^{2}\end{array}& \begin{array}{ll}t& 1\end{array}\end{array}\right] \left[\begin{array}{*{20}c} { - 1} & 3 & { - 3} & 1 \\ 3 & { - 6} & 3 & 0 \\ { - 3} & 0 & 3 & 0 \\ 1 & 4 & 1 & 0 \\ \end{array}\right] \left[\begin{array}{l}{P}_{i}\\ {A}_{i,1}\\ \begin{array}{l}{A}_{i+1,0}\\ {P}_{i+1}\end{array}\end{array}\right],$$

25 $${r}_{3i}\left(t\right)=\frac{1}{6}\left[\begin{array}{ll}\begin{array}{ll}{t}^{3}& {t}^{2}\end{array}& \begin{array}{ll}t& 1\end{array}\end{array}\right] \left[\begin{array}{*{20}c} { - 1} & 3 & { - 3} & 1 \\ 3 & { - 6} & 3 & 0 \\ { - 3} & 0 & 3 & 0 \\ 1 & 4 & 1 & 0 \\ \end{array}\right] \left[\begin{array}{l}{A}_{i,1}\\ {A}_{i+1,0}\\ \begin{array}{l}{P}_{i+1}\\ {A}_{i+1,1}\end{array}\end{array}\right].$$

Since the basis function of the traditional B-spline curve has a coefficient of 1/6, h = 1/6 is taken in the expression of the improved B-spline curve fitting method. According to Eqs. (21) and (22), all type value points are represented by original type value points. Taking P_1_, P_2_, P_3_, and P_4_ points as examples, the equation of the fitting curve expressed in the form of a matrix is as follows:

26 $${r}_{1}\left(t\right)=\frac{1}{6}\left[\begin{array}{ll}\begin{array}{ll}{t}^{3}& {t}^{2}\end{array}& \begin{array}{ll}t& 1\end{array}\end{array}\right] \left[\begin{array}{*{20}c} {\frac{1}{3}} & -{\frac{5}{6}} & {\frac{2}{3}} & -{\frac{1}{6}} \\ 0 & 0 & 0 & 0 \\ { - 1} & 0 & 1 & 0 \\ 0 & 6 & 0 & 0 \\ \end{array}\right] \left[\begin{array}{l}{P}_{1}\\ {P}_{2}\\ \begin{array}{l}{P}_{3}\\ {P}_{4}\end{array}\end{array}\right]=T{B}_{1}P,$$

27 $${r}_{2}\left(t\right)=\frac{1}{6}\left[\begin{array}{ll}\begin{array}{ll}{t}^{3}& {t}^{2}\end{array}& \begin{array}{ll}t& 1\end{array}\end{array}\right] \left[\begin{array}{*{20}c} -{\frac{1}{2}} & {\frac{3}{2}} & -{\frac{3}{2}} & {\frac{1}{2}} \\ 1 & -{\frac{5}{2}} & 2 & -{\frac{1}{2}} \\ {0} & -{\frac{5}{2}} & 3 & -{\frac{1}{2}} \\ -{\frac{2}{3}} & 5{\frac{1}{6}} & {\frac{5}{3}} & -{\frac{1}{6}} \\ \end{array}\right] \left[\begin{array}{l}{P}_{1}\\ {P}_{2}\\ \begin{array}{l}{P}_{3}\\ {P}_{4}\end{array}\end{array}\right]=T{B}_{2}P,$$

28 $${r}_{3}\left(t\right)=\frac{1}{6}\left[\begin{array}{ll}\begin{array}{ll}{t}^{3}& {t}^{2}\end{array}& \begin{array}{ll}t& 1\end{array}\end{array}\right] \left[\begin{array}{*{20}c} {\frac{1}{6}} & - {\frac{2}{3}} & {\frac{5}{6}} & - {\frac{1}{3}} \\ - {\frac{1}{2}} & 2 & - {\frac{5}{2}} & 1 \\ {\frac{1}{2}} & -3 & {\frac{5}{2}} & 0 \\ -{\frac{1}{6}} & {\frac{5}{3}} & 5{\frac{1}{6}} & -{\frac{2}{3}} \\ \end{array}\right] \left[\begin{array}{l}{P}_{1}\\ {P}_{2}\\ \begin{array}{l}{P}_{3}\\ {P}_{4}\end{array}\end{array}\right]=T{B}_{3}P.$$

Then curve fitting can be carried out section by section according to Eq. (26) ~ Eq. (28) to determine the fitting curve obtained by the improved B-spline curve fitting algorithm. Since the basis function of the improved B-spline curve fitting algorithm is based on the basis function of the B-spline curve fitting algorithm, the interpolation curve not only has the advantages of the local, continuous and convex hull of the traditional B-spline curve but also improves the curve fitting accuracy through all the original type value points.

### GDPSO-RBF neural network model

**Figure 9:**
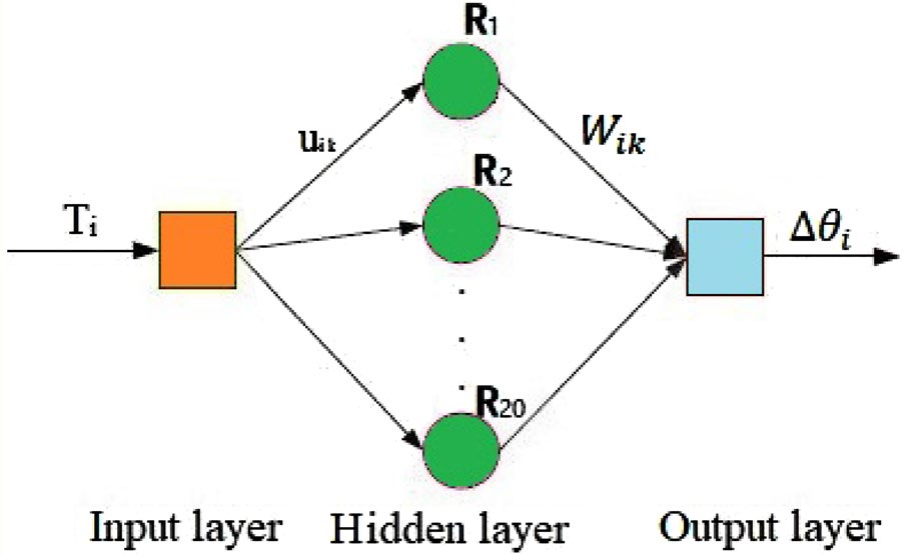
Error compensation model.

The RBF neural network algorithm has strong nonlinear mapping ability and generalization ability. Its model includes an input layer, at least one hidden layer, and one output layer. This algorithm does not need to consider the linearity or nonlinearity of the compensation object and only focuses on the input and output conditions. The approaching equation effect of the RBF neural network is better when the system is stable, and the influence of environmental factors is small. Especially when there are many sample points and the compensation accuracy is high^28,29^. Therefore, a radial basis function (RBF) neural network can be used to realize continuous compensation. As shown in Fig. 9, the RBF neural network for the error compensation is a three-layer unidirectional propagation network. The loading torque and the angle measurement error are used as the learning samples of the input and output layers, respectively, for training.

The input layer neurons directly map the input data to the hidden layer neurons. The dimension of the center vector is equal to the dimension of the input vector. The dimension of the center vector on the hidden layer neuron is equal to twenty, which is verified to be converged fastest through repeated debugging. The Gaussian function is selected as the basis function on the hidden layer node of the RBF neural network. The role of the Gaussian function in the RBF neural network model is to activate samples:

29 $${R}_{k}={e}^{-\frac{\Vert {u}_{ik}{T}_{i}-{c}_{k}\Vert }{2{\sigma }_{k}^{2}}}={e}^{-\frac{\Vert \sqrt{{{\Delta \theta }_{1}}^{2}+{{\Delta \theta }_{2}}^{2}+{{\Delta \theta }_{3}}^{2}+{{\Delta \theta }_{4}}^{2}+{{\Delta \theta }_{5}}^{2}+{{\Delta \theta }_{6}}^{2}}-{c}_{k}\Vert }{2{\sigma }_{k}^{2}}.}$$

In the equations above, ${T}_{i}$ represents the input sample, ${c}_{k}$ represents the central vector of the hidden layer neuron whose dimension is equal to the input sample dimension, ${\sigma }_{k}$ indicates the width of hidden layer neurons, and ‖${u}_{ik}{T}_{i}-{c}_{k}$‖ represents the Euclidean distance between the center vector and the sample. ${c}_{k}$, ${\sigma }_{k}$ and ${W}_{ik}$ are called mathematical parameters of the neural network model. When the value of ${\sigma }_{k}$ is constant, the value of the RBF function reaches the maximum at ‖${u}_{ik}{T}_{i}-{c}_{k}$‖ where it is zero and decays rapidly to zero as the distance increases. Therefore, when the width ${\sigma }_{k}$ and the center vector ${c}_{k}$ are determined, the RBF function has the characteristics of local response to sample ${T}_{i}$. The final prediction result for a single sample is a linear combination of the outputs of all hidden layer nodes:

30 $${\Delta \theta }_{i}=\sum_{k=1}^{20}{W}_{ik}{R}_{k}.$$

In the equations above, ${W}_{ik}$ denotes the output weights from ${R}_{k}$ to ${\Delta \theta }_{i}$. Equation (30) can calculate the target prediction result according to the known input sample data, so Eq. (30) is the neural network prediction model. The difference between the result vector and the expected output vector is the overall sample fitting error:

31 $$e=\sum_{k=1}^{20}\left({\Delta \theta }_{ik}-{\Delta {\theta }^{{\prime}}}_{ik}\right),$$

Here, ${\Delta {\theta }^{{\prime}}}_{ik}$ represents the result vector of the output layer.

It can be seen that the RBF neural network model is an accurate mathematical model with uncertain parameters. When the structure and mathematical parameters of the network are determined, the output results of the same input sample will not change. Therefore, the design process of the RBF neural network model is the process of determining the structure and mathematical parameters: the process of adjusting the center vector and width is the process of selectively responding to the samples. Its essence is the reasonable distribution of the center vector in the overall sample space. From a mathematical point of view, the adjustment of ${W}_{ik}$ can be understood as a linear equation-solving process.

After initializing the parameter values, the traditional RBF model uses sample clustering or gradient descent to solve the structure and mathematical parameters. This highly targeted, accurate mathematical algorithm often leads to the model’s inability to find the optimal global solution^30^. In this paper, gradient descent and particle swarm optimization (GDPSO) methods are used to solve the center vector, hidden layer neuron width, and hidden layer neuron weight, which can not only improve the global search performance of the algorithm but also give consideration to the operation speed and local optimization performance. The GDPSO is a swarm intelligence algorithm. Each particle in the population corresponds to a candidate solution to a problem. Affected by both learning and memory, the particle transitions to complete global optimization tasks in the multi-dimensional solution space. The basic process is as follows:

It is assumed that the solution space dimension is d and the particle population size is s. The particle position is substituted into the problem objective function to solve the corresponding fitness of the particle, the individual historical optimal fitness and the optimal global fitness are recorded, and the speed of particle i is given. Then, when the fitness value does not meet the requirements, the position and speed of particles are iteratively calculated. The iterative formula of speed and position is shown in Formula (32) and Formula (33):

32 $${v}_{ij}^{m+l}=w{v}_{ij}^{m}+{c}_{1}{r}_{1}\left({p}_{ij}^{m}-{x}_{ij}^{m}\right)+{c}_{2}{r}_{2}\left({g}_{j}^{m}-{x}_{ij}^{m}\right),$$

33 $${x}_{ij}^{m+l}={x}_{ij}^{m}+l{v}_{ij}^{m+l},$$

In the above equations, m is the number of iterations, j represents the jth element in the vector, ${\mathrm{x}}_{\mathrm{ij}}^{\mathrm{m}}$ is the location of particle i in space, ${\mathrm{v}}_{\mathrm{ij}}^{\mathrm{m}}$ is the speed of particle i in space, ${\mathrm{p}}_{\mathrm{ij}}^{\mathrm{m}}$ and ${\mathrm{g}}_{\mathrm{j}}^{\mathrm{m}}$ are defined as local and global fitness, respectively. ${\mathrm{c}}_{1}$ and ${\mathrm{c}}_{2}$ are acceleration coefficients, ${\mathrm{r}}_{1}$ and ${\mathrm{r}}_{2}$ are random numbers in the interval [0,1], and $\mathrm{w}$ is the weight.

34 $$w={w}_{max}-\left({w}_{max}-{w}_{min}\right)\times \frac{m}{{m}_{max}},$$

In the equation above, ${\mathrm{m}}_{\mathrm{max}}$ is the maximum number of iterations, ${\mathrm{w}}_{\mathrm{max}}$ and ${\mathrm{w}}_{\mathrm{min}}$ represents the maximum and minimum weights, respectively.

The traditional particle swarm optimization algorithm does not deal with discrete optimization problems. The Gradient descent (GD) algorithm is used to find the optimal global solution of the RBF neural network and avoid falling into local optimum or non-convergence. The GD algorithm is a supervised optimization learning algorithm. Unlike the general heuristic algorithm, the basic theory of GD is the principle of calculus. It finds the direction in which the objective function value can produce the maximum change in the multi-dimensional solution space by solving the derivative and moves closer to that direction with a specific step size to achieve the goal of decreasing or raising the objective function.

The specific method is as follows: the objective function is maximized or minimized, and the objective function is required to calculate the partial derivatives of all independent variables. The obtained partial derivatives are scientifically reduced as the step size for adjusting the corresponding independent variables, and the iterative operation is carried out until the objective function value meets the problem requirements. The word “scientifically” here means following the simple line-search guidelines.

Aiming at the parameter optimization of the RBF neural network model and considering the practicability of optimization efficiency, the objective function of the GD algorithm is obtained as follows:

35 $$E=\frac{1}{2}\sum_{k=1}^{20}{\left({\Delta \theta }_{ik}-{\Delta {\theta }^{{\prime}}}_{ik}\right)}^{2},$$

Particle swarm optimization (PSO) with gradient affects the update of particle velocity by introducing gradient information. Each particle is updated probability p according to the negative gradient. Moreover, the PSO is updated according to the probability of 1-P. In this way, when the optimal information of the group is stagnant, some group particles can be reinitialized to keep the group’s activity and reduce the possibility of the group falling into the local optimum. At the same time, we can adjust w, c_1_, and c_2_ to thoroughly search each area early and accelerate the convergence later. Some other mechanisms can be introduced, such as random factors, boundary changes in speed and position, etc. Combine with other optimization algorithms: genetic algorithms, simulated annealing algorithms, etc., to help particles jump out of local optimum and control convergence speed.

The GDPSO algorithm steps are as follows:

- Step 1: Determine relevant parameters, particle swarm size, the maximum number of iterations, linear inertia weight, acceleration coefficient, target accuracy, RBF hidden layer node number, and gradient descent selection probability.
- Step 2: Determine the initial distribution interval of particle position and velocity, randomly initialize the particle position and velocity matrix, and rank the position and velocity parameters in the order of neuron-width center-vector weight.
- Step 3: Determine the evaluation function of particles.
- Step 4: Substitute the existing particles into the evaluation function to obtain the evaluation value, update the particles’ historical and global extreme value, and judge whether the evaluation value < ε or k > iter-max is met. If yes, the algorithm ends and records the optimal position of the existing particles; If not, turn to step 5.
- Step 5: The particles are selected by probability. GD iterates the selected particles, and the remaining particles are iterated by Formula (32) and Formula (33).
- Step 6: k = k + 1, turn to step 4.

The realization steps of the GDPSO-RBF model include neural network construction, training, and prediction. Three (or more than three) groups of one thousand points evenly distributed in the loading range obtained from the angle measurement error calibration are taken as the training sample. Another three (or more than three) groups of one thousand points evenly distributed in the loading range obtained from the angle measurement error calibration are taken as the testing sample. The training is mainly to assign the optimal weight and threshold obtained by the GDPSO algorithm to the RBF neural network as the initial weight and point of the network. The training samples are substituted into the network for training and testing. If the actual output of 1000 test samples is consistent with the expected result, the network’s generalization ability is good, and the training is completed. Finally, the angle measurement errors in the loading range are predicted. The above three-step process can be realized using the newrb-function, train-function, and sim-function provided by the neural network toolbox in MATLAB.

### IBSCF-GDPSO-RBF method

As described above, the loading stability limits the number of sampling points. Traditional numerical compensation methods, such as the polynomial fitting and the B-spline curve fitting, are used to fit the angle measurement error caused by deformation. However, it is challenging to model nonlinear data or data features with correlation polynomial regression, and it is challenging to express highly complex data well. The fitted angle measurement error model cannot fully reflect the characteristics of the relationship between the angle measurement error and the loading torque due to the limitation of the sample points’ number. So the compensation effect is limited.

On the contrary, the RBF neural network has the following advantages: 1. Its multi-layer nonlinear structure can express very complex nonlinear relations. 2. The flexibility of its model makes us not need to care about the data structure. 3. The more data, the better the network performance. Because of this, this paper combines the improved B-spline curve fitting method with the GDPSO-RBF neural network method. Based on the IBSCF-GDPSO-RBF method, compensation accuracy for the angle measurement error is further improved. The specific implementation steps of the IBSCF-GDPSO-RBF combination fitting method are as follows.

- Step 1: Measuring multiple groups of angle measurement errors caused by the combined deformation of the MSIS (or MSOS) with different initial loading torque values. The instrument deformation calibration device is installed on the instrument, and the initial loading torque value is 0 Nm (± 0.01 Nm). Then the loading torque value of MSIS is adjusted to 0.05 Nm, 0.1 Nm, 0.15 Nm, 0.15 Nm, 0.2 Nm $\cdots$, and 50 Nm (or 2 Nm, 4 Nm, 6 Nm, 8 Nm, 10 Nm $\cdots$, and 2000 Nm for the MSOS). One thousand points $({T}_{i},{\Delta \theta }_{i} )$ in the whole torque loading range (*i* = 1, 2, …, 1000) are obtained again. The experiment is repeated three times to obtain the first group angle measurement errors caused by the combined deformation of the MSIS (or MSOS).
- Step 2: Fitting the relationship curve. The angle measurement error-loading torque relationship model $\Delta \theta ( T)$ is fitted based on the angle measurement error and the loading torque using the improved B-spline curve fitting method.
- Step 3: Multi-group data prepossessing. The angle measurement error and the loading torque are used as the benchmark. Selecting the loading torque interval $({T}_{i}^{1},{\Delta \theta }_{i}^{1} )$ within which the angle measurement error-loading torque relationship model $\Delta \theta ( T)$ of the angle measurement error value has a stable variation trend. The loading torque quantity $({T}_{i}^{2},{\Delta \theta }_{i}^{2})$, $({T}_{i}^{3},{\Delta \theta }_{i}^{3} )$ the other two groups are selected. Then the adjustment coefficients ${a}_{i}^{2}$ and ${a}_{i}^{3}$ of the other two groups can be obtained using the fitting value $\Delta \theta \left( {T}_{i}^{2}\right), \Delta \theta ( {T}_{i}^{3})$ of the angle measurement error values, subtract the value ${\Delta \theta }_{i}^{2}$ and ${\Delta \theta }_{i}^{3}$ of the actual angle measurement error, that is:
  36 $${a}_{i}^{2}=\Delta \theta \left( {T}_{i}^{2}\right)-{\Delta \theta }_{i}^{2},$$
  37 $${a}_{i}^{3}=\Delta \theta \left( {T}_{i}^{3}\right)-{\Delta \theta }_{i}^{3}.$$
  Then ${a}_{i}^{2}$ and ${a}_{i}^{3}$ are added to the value ${\Delta \theta }_{i}^{2}$ and ${\Delta \theta }_{i}^{3}$ to calculate the angle measurement error value according to formula (38)–(40), the final angle measurement error value could be correlated.
  38 $${{\Delta \theta }_{i}^{1}}^{^{\prime}}={\Delta \theta }_{i}^{1},$$
  39 $${{\Delta \theta }_{i}^{2}}^{^{\prime}}={\Delta \theta }_{i}^{2}+{a}_{i}^{2},$$
  40 $${{\Delta \theta }_{i}^{3}}^{^{\prime}}={\Delta \theta }_{i}^{3}+{a}_{i}^{3}.$$
- Step 4: The prepossessing data of the angle measurement error-loading torque $({T}_{i},{\Delta \theta }_{i})$ is substituted with the GDPSO-RBF neural network. The final relationship model between the angle measurement error-loading torque is obtained. The hidden layer node number is obtained according to Eq. (29). Furthermore, the effect is estimated according to the fitted relationship model.

In testing the relevant parameters of the reducer, the corresponding angle error compensation value is calculated according to the loading torque value brought into the angle measurement error-loading torque relationship model and compensated for the final angle measurement result. Then the actual torsion angle of the reducer can be obtained using the angle measurement result minus the angle error compensation value. In this way, the compensation for the angle measurement error caused by the deformation of the transmission chain shafting in the MSIS and MSOS can be realized, respectively. After the error compensation, the torsion angle of the reducer under any torque is:

41 $${\theta }^{^{\prime}}=\theta -\Delta \theta .$$

Finally, the stiffness and other relevant parameters can be calculated according to the torsion angle under any torque after error compensation.

## Experiment results

**Figure 10:**
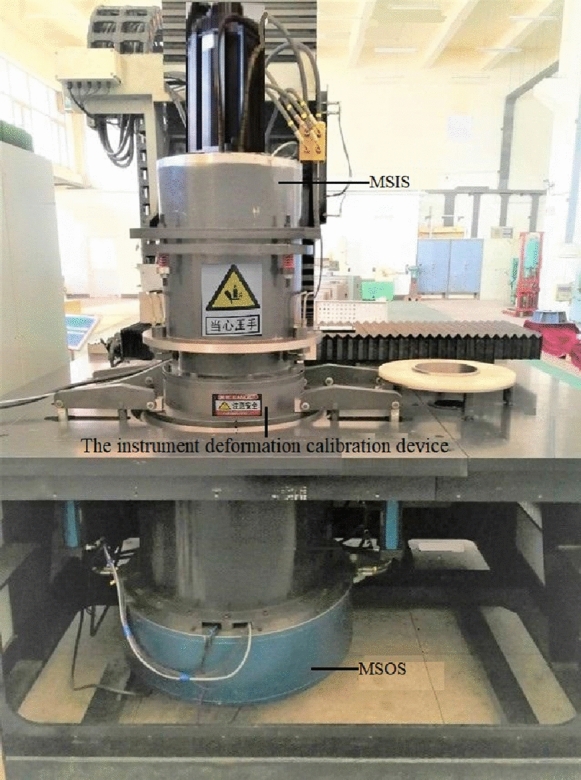
Calibration experiment of the angle measurement error caused by the deformation of the instrument.

According to the angle measurement error calibration and compensation method proposed in the third and fourth sections, a series of experiments were designed to determine the accuracy of the calibration and compensation of angle measurement error and the accuracy of the RV reducer deformation measurement based on the proposed method^31^. First, the error calibration experiment under different loading torques was carried out on the precision reducer detector, as shown in Fig. 10. The list of “angle measurement error-loading torque” of the MSIS and MSOS is obtained. Then, the angle measurement error-loading torque relationship curve is fitted using the IBSCF-GDPSO-RBF method. Besides, the MSIS (or MSOS) motor is used again to synchronously load the torque to the instrument deformation calibration device and the MSIS (or MSOS), and the error compensation method described in this paper is used to compensate for the angle measurement error. This step is carried out to verify the effect of error compensation. Last but not least, the TA was installed on the instrument, and the stiffness of the reducer was tested based on the error compensation method.

Modrol Electric CO., Ltd made the servo motor (or torque motor) used in the MSIS (or MSOS). In the experiment, the MSIS (or MSOS) was driven by the servo motor (or torque motor). The servo motor (or torque motor) was controlled to work in the torque mode to ensure loading stability during the measurement. The model of the servo motor was SMS15-42P2C. The loading range of the servo motor was 50 Nm, and the loading accuracy of the servo motor was 0.1% on the full scale. The model of the torque motor was SMC35-42T2A. The loading range of the torque motor was 2000 Nm, and the loading accuracy of the torque motor was 0.1% on the full scale.

The HBM company produced the torque transducer used in the instrument. The model of the torque transducer was T40B. The torque transducer measuring range in the MSIS (or MSOS) was 0–50 Nm (or 0–2000 Nm), and its measuring accuracy was 0.1% at full scale. Angular measurement systems in MSIS and MSOS adopt absolute optical encoders produced by the Renishaw company. The model of the absolute optical encoder was RESA-30U-S-A3000-B. Its measuring accuracy is 0.96″. A PXIe acquisition system produced by the National Instruments company was used to collect the angle signal and the torque signal at a rate of 25 k samples per second. The accuracy of torque signal acquisition was 0.01% on the full scale. The accuracy of angle signal acquisition was 0.01″.

### The angle measurement error calibration

The instrument deformation calibration device calibrates the angle measurement error caused by instrument deformation. Its functional objective is to separate the angle measurement error and the deformation of the measured reducer under various torques. The basic idea is to replace the position of the measured reducer with an instrument deformation calibration device in the measuring shafting of the instrument and use the reading of the angle measurement system to represent the angle measurement error caused by the instrument deformation under a specific loading torque. The experiment of using the instrument deformation calibration device designed in this paper to calibrate the angle measurement error of the instrument shafting under different loading torques should be carried out according to the following procedures:

- ① Install the instrument deformation calibration device between the MSIS and MSOS, and position and compress it with the barrel-shaped bench at the MSIS and MSOS through the seam, replacing TA. The calibration device is connected to the measuring shaft system of the MSIS and MSOS.
- ② The hydraulic cylinder drives the rectangular spline sleeve of the MSIS and MSOS. Then, the shafting of the measuring system at the MSIS (or MSOS) is in the transmission state, and the shafting of the measuring system at the MSOS (or MSIS) is in the disconnection state. 1% FS (about 0.5 N · m) torque is applied to the motor of the measuring system at the MSIS (or MSOS). Then it can be ensured that the spline shaft at the input (or output) end of the calibration device is fully fitted with the spline sleeve of the MSIS (or MSOS) at this time, and all intermediate connecting structures are slightly deformed under force, thus eliminating the gap.
- ③ The motor of the MSIS (or MSOS) is slowly loaded in the forward and reverse directions (clockwise and counterclockwise) until the maximum measured torque is reached. The critical torque of the required specific reducer is converted into the MSIS (or MSOS) torque value. When the torque value measured by the MSIS (or MSOS) reaches the critical torque value, the readings of the instrument’s circular grating angle measuring system and the corresponding torque sensor of the MSIS (or MSOS) are recorded in this process.
- ④ The step ③ is repeated three times in sequence. Take the average of the three measurement results as the “angle measurement error-loading torque” of the MSIS (or MSOS) measurement point. Then, the list of “angle measurement error-loading torque” is obtained.

**Figure 11:**
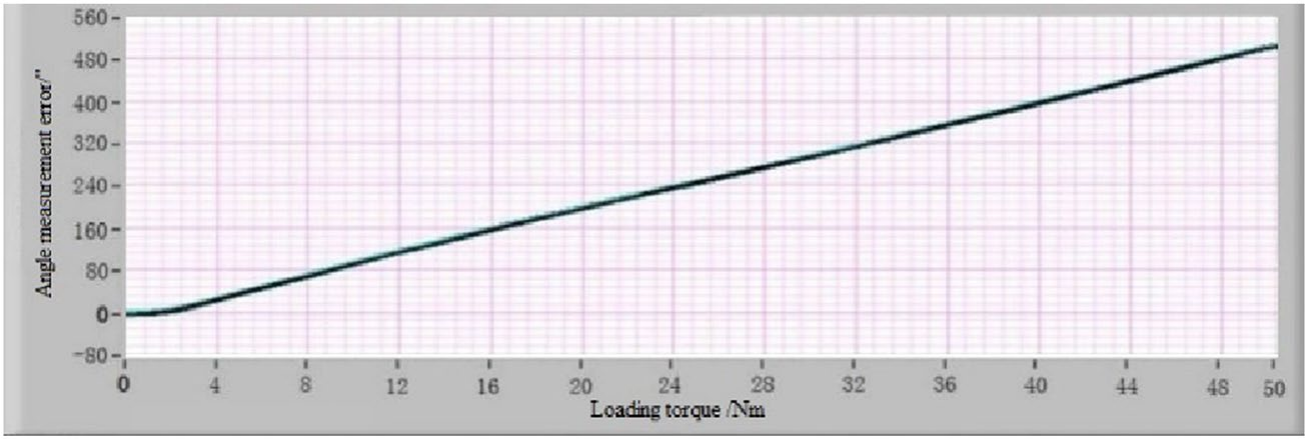
The angle measurement error-loading torque relationship curve of MSIS.

**Figure 12:**
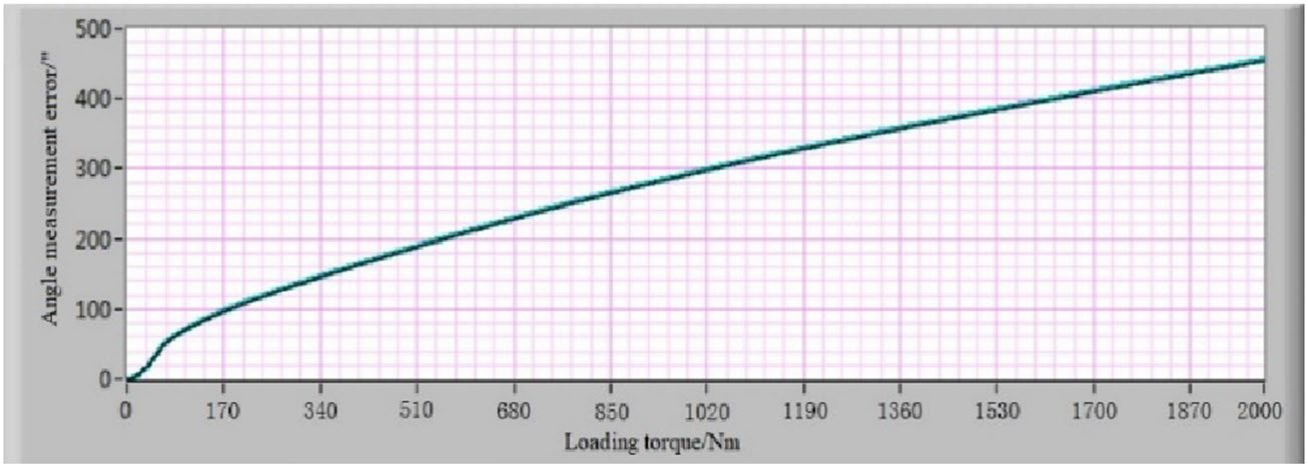
The angle measurement error-loading torque relationship curve of MSOS.

After obtaining the list of “angle measurement error-loading torque” of the MSIS and MSOS, the angle measurement error-loading torque relationship curve can be fitted according to it using the IBSCF-GDPSO-RBF method. The instrument deformation calibration device is installed between the MSIS and MSOS to verify the effect of the error compensation method described in this paper. The error calibration experiment under different loading torques was carried out according to the above procedure. The list of “angle measurement error-loading torque” is obtained, and the angle measurement error-loading torque curve is fitted according to the list. The angle measurement error-loading torque curves of the MSIS and MSOS using the IBSCF-GDPSO-RBF method are shown in Figs. 11 and 12. The compensation values of angle measurement error of the MSIS and MSOS at any torque are obtained from these curves to compensate for the measured torsion angle of the reducer at any torque.

### Verification of the angle measurement error calibration and error compensation method

**Figure 13:**
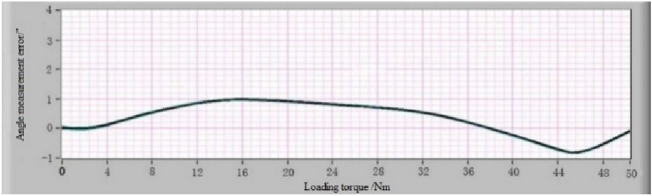
Errors of MSIS after compensation using the IBSCF-GDPSO-RBF method.

**Figure 14:**
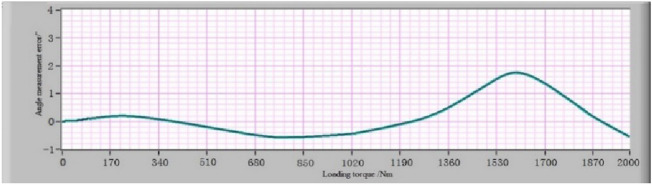
Errors of MSOS after compensation using the IBSCF-GDPSO-RBF method.

**Figure 15:**
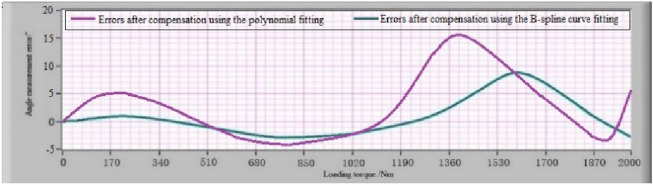
Errors of MSOS after compensation using the polynomial fitting and the B-spline curve fitting method.

Since the angle measurement error-loading torque curves of the MSIS and MSOS are obtained, then the motor of the MSIS and MSOS is used again to load the instrument deformation calibration device to verify the effect of the error compensation method described in this paper. According to the formula (41), the error compensation was realized using the angle measurement result minus the angle error compensation value calculated according to the loading torque value brought into the angle measurement error-loading torque relationship curve^31^. The angle measurement errors caused by the deformation of the MSIS and MSOS after error compensation are represented in Fig. 13 and 14, respectively. The results show that the maximum angular measurement error of the MSIS is ± 1″, and the maximum angular measurement error of the MSOS is ± 2″. The angular measurement accuracy of the MSIS and the MSOS can reach ± 2″ through error compensation. For comparison, the polynomial fitting and the B-spline curve fitting were used to compensate for the angular measurement error, respectively. The angular measurement error of the MSOS after compensation is shown in Fig. 15.

Then, the TA was installed on the instrument, and the torsional stiffness of the RV-40E reducer was tested to verify the MSIS and MSOS error compensation effect using the IBSCF-GDPSO-RBF method. Torsional stiffness is one of the most critical static parameters of the reducer, which is used to measure the ability of the reducer to resist torsional deformation under the action of torque and directly or indirectly affects the positioning accuracy and bearing capacity of industrial robots in work. The essential requirement of the torsional stiffness test is to fix the input end of the reducer, gradually load the torque on the output end from the free state to the rated torque or set value, then reverse load to the rated torque or set value, and then return to the initial state. In this process, the corresponding values of the angle at the output end and the loaded torque are recorded synchronously in real time. In the measurement process, since the reducer is fixed at the input end, the angle at the input end should be zero. It means if the input end is fixed well enough, the input sensors (torque & angle) are not needed. If the measurement result of the angular sensor at the input end is not zero, the torsional deformation of the reducer should be obtained using the angle at the output end minus the output end conversion value of the input end angular sensor measurement result. This paper uses the subsection method to calculate the torsional stiffness data. According to the experimental requirements, the least square method is used to fit the slope k of the straight line for each curve segment, and its reciprocal is the torsional stiffness of the measured reducer corresponding to this segment. Assume that the segmentation space is -100% rated torque to -a% rated torque, -a% rated torque to + a% rated torque, and + a% rated torque to + 100% rated torque. The slope value of each curve can be obtained as ${k}_{1}$, ${k}_{2}$, ${k}_{3}$. Then the torsional stiffness of -a% rated torque to + a% rated torque segment is:

42 $${K}_{2}=\frac{1}{{k}_{2}},$$

The torsional stiffness of -100% rated torque to -a% rated torque segment and a% rated torque to 100% rated torque segment are both:

43 $${K}_{1}=(1/{k}_{1}+1/{k}_{3})/2,$$

Three independent repeated experiments were conducted on the reducer. The torsional stiffness of the RV-40E reducer was calculated according to the reducer deformation under different torques measured in the three independent repeated experiments. The calculation results of the torsional stiffness are shown in Table 1.

**Table 1:** The torsional stiffness and backlash of the RV-40E reducer.

Results	Torsional stiffness(Nm/”)	
	${K}_{1}$	${K}_{2}$
The first time	15.51	1.04
The second time	15.52	1.06
The third time	15.50	1.05

It can be figured out from Table 1 that the measurement repeatability error of the reducer torsional stiffness is within ± 0.01Nm/angular second. After the angle measurement error compensation, the measurement accuracy of the reducer torsional stiffness is high, meeting the requirements of high-precision measurement.

## Conclusion

The features of the angle measurement error caused by instrument deformation are studied based on the structure of the high-precision detector. Based on the features, a new method of calibration and compensation of the angle measurement error based on the improved B-spline curve fitting-gradient descent and particle swarm optimization -radial basis function neural network (IBSCF-GDPSO-RBF) method is proposed to eliminate the influence of the torsional deformation of the instrument. The problem that the change of instrument deformation affects the angle measurement accuracy of the instrument is solved. It can be carried out that the angle measurement error caused by the instrument deformation after compensation is within ± two angular seconds. The contribution of this paper is that the method calibrates and compensates for the angle measurement error based on the IBSCF-GDPSO-RBF method, which is not limited to the background of measuring the torsional deformation of the RV reducer. It provides a reference for measuring and evaluating the actual torsional rigidity of the RV reducer under any load.

## Data Availability

The data used to support the findings of this study can be accessed from the corresponding author upon request.

## References

[1] Chu XY (2019). The method of selective assembly for the RV reducer based on genetic algorithm. Proc. Inst. Mech. Eng. C J. Mech. Eng. Sci..

[2] Pham AD, Ahn HJ (2018). High precision reducers for industrial robots driving 4th industrial revolution: State of arts Analysis, Design, Performance Evaluation, and Perspective. Int. J. Prec. Eng. Manuf.-Green Technol..

[3] Sun X, Han L, Ma KW, Li LY, Wang J (2018). Lost motion analysis of CBR reducer. Mech. Mach. Theory.

[4] Taghirad HD, Bélanger PR (1998). Modeling and parameter identification of harmonic drive systems. J. Dyn. Syst. Meas. Control.

[5] Deng FK (2019). Life calculation of angular contact ball bearings for industrial robot RV reducer. Ind. Lubr. Tribol..

[6] Pham A, Ahn H (2018). High precision reducers for industrial robots driving 4th industrial revolution: state of arts, analysis, design, performance evaluation and perspective. Int. J. Prec. Eng. Manuf.-Green Technol..

[7] Hang XU (2019). Dynamic measurement of the lost motion of precision reducers in robots and the determination of optimal measurement speed. J. Adv. Mech. Des. Syst. Manuf..

[8] Iwasaki, M., Nakamura H. Vibration suppression for angular transmission errors in harmonic drive gearings and application to industrial robots. Preprints at *19th World Congress, The International Federation of Automatic Control* 6831–6836 (2014).

[9] Sun YG, Zhao XF, Jiang F, Zhao L, Liu D, Yu GB (2014). Backlash analysis of RV reducer based on error factor sensitivity and Monte-Carlo Simulation. Int. J. Hybrid Inf. Technol..

[10] Bin Z, wei Q, Jin L (2012). Simulation and analysis of dynamical transmission precision of 2K-V cycloidal pin gear reducer based on multibody system dynamics. Adv. Mater. Res..

[11] Fu, W.-b., Ai, C.-s., Tang, C.-l., Yang, Y.-s., Li, G.-p., Zhao, H.-h. RV reducer dynamic performance test bed design in closed power flow. In *4th International Conference on Sensors, Mechatronics and Automation* 666–669 (2016).

[12] Tran TL, Pham AD, Ahn HJ (2016). Lost motion analysis of one stage cycloid reducer considering tolerances. Int. J. Precis. Eng. Manuf..

[13] Hejny, S. W. Design of a Harmonic Drive Test Apparatus for Data Acquisition and Control. M.S Thesis, Rice Univ. (1997).

[14] Xu, J., Sheng, G. Theoretical Calculation and Simulation Analysis of No-load Torque of Main Reducer. In *2017 International Conference on Applied Mathematics, Modeling and Simulation (AMMS 2017)* (2017).

[15] Kim KH, Lee CS (2009). Torsional Rigidity of a Two-stage Cycloid Drive. Trans. Kore. Soci. Mech. Eng. A..

[16] Park MW, Jeong JH, Ryu JH (2007). Development of speed reducer with planocentric involute gearing mechanism. J. Mech. Sci. Tech..

[17] Yang YH (2019). Response Sensitivity to design parameters of RV reducer. Chin. J. Mech. Eng..

[18] Park YK, Kim MS, Lee JT (2009). Torque traceability examination of calibration laboratories in Korea. Measurement.

[19] Wang RY (2019). Meshing efficiency analysis of modified cycloidal gear used in the RV reducer. Tribol. Trans..

[20] Xu H, Liu X (2014). Analysis for assembly dimension chain of RV reducer. Appl. Mech. Mater..

[21] Wang ZQ, Jin M (2005). Analysis of shear strain under large torsion deformation. J. North. Jiaotong Univ..

[22] Jia HK, Yu LD, Zhao HN (2019). A new method of angle measurement error analysis of rotary encoders. Appl. Sci..

[23] Saygun A, Omurtag MH, Orakdogen E (2007). A simplified solution of the torsional rigidity of the composite beams by using FEM. Adv. Struct. Eng..

[24] Sigmund O (1995). Tailoring materials with prescribed elastic properties. Mech. Mater..

[25] Yu Z, Qiu ZR, Li H (2022). Measuring the no-load running torque of RV reducer based on the SVD and MCSA. Measurement.

[26] Barrot A, Paredes M, Sartor M (2006). Determining both radial pressure distribution and torsional stiffness of involute spline couplings. Proc. Inst. Mech. Eng. Part C..

[27] Goodno BJ (2016). Mechanics of Materials.

[28] Ogidi OO, Barendse PS, Khan MA (2018). Measuring fault indicators in electric machines—Learning experience. IEEE Instrum. Meas. Mag..

[29] Alippi C, Piuri V, Scotti F (2001). Accuracy versus complexity in RBF neural networks. IEEE Instrum. Meas. Mag..

[30] Yang J, Zhou J, Liu L (2009). A novel strategy of pareto-optimal solution searching in multi-objective particle swarm optimization (MOPSO). Comput. Math. Appl..

[31] Zhen Yu, Qiu Z, Li H, Xue J, Wenchuan Hu, Wang C (2022). Design and calibration of torque measurement system of comprehensive performance test instrument of industrial robot reducer. Comput. Intell. Neurosci..

